# Bone marker reference range study: a comparison of the manufacturer’s reference range and laboratory healthy volunteer results to patients with rheumatoid arthritis

**DOI:** 10.1186/1471-2474-14-S1-A3

**Published:** 2013-02-14

**Authors:** Gillian Wheater, Mohsen Elshahaly, Stephen P Tuck, John Drury, Jaap M van Laar

**Affiliations:** 1 Biochemistry Department, The James Cook University Hospital, Middlesbrough, Cleveland, TS4 3BW, UK; 2 Musculoskeletal Research Group, Institute of Cellular Medicine, Newcastle University, Newcastle-upon-Tyne, NE1 7RU, UK; 3 Rheumatology Department, The James Cook University Hospital, Middlesbrough, Cleveland, TS4 3BW, UK

## Background

Biochemical markers of bone turnover have been used in research for a long time and are now being recognised as helpful tools in the clinical management of bone disease. It is important to establish robust reference values for the interpretation of these markers. In addition to standardising pre-analytical variability it is unclear whether manufacturer’s ranges take into account global differences between subjects and so each laboratory should investigate the transferability of the expected values to its own patient population and if necessary determine its own reference ranges.

## Materials and methods

Serum samples from 70 healthy volunteers were analysed for biochemical markers of bone formation (procollagen type I amino-terminal propeptide [P1NP] and osteocalcin [OC]) and bone resorption (beta carboxyterminal cross-linking telopeptide of bone collagen [βCTX] on the Roche Elecsys 2010. The results were compared to the manufacturer’s reference range and to 46 samples from patients with severe refractory rheumatoid arthritis prior to treatment with rituximab.

## Results

We found substantial inter-person variability in all of the biomarkers and differences between the manufacturer and healthy control ranges (Table 1).

**Table 1:** Comparison of bone marker results between manufacturer, healthy controls and rheumatoid arthritis patients

	Manufacturer			Healthy controls			Rheumatoid arthritis		
	
	Pre^c^	Post^d^	Male	Pre^c^	Post^d^	Male	Pre^c^	Post^d^	Male
**βCTX^a^** **ng/L**	**299** **±274**	**556** **±452**	**300** **±284**	**192** **±196**	**199** **±264**	**325** **±382**	**139** **±184**	**354** **±572**	**337** **±410**
**P1NP^b^** **µg/L**	**27.8** **15-59**	**37.1** **16-74**	**No data**	**31.9** **22-58**	**32.6** **18-66**	**51.5** **22-79**	**30.1** **11-50**	**39.1** **12-73**	**40.5** **13-81**
**OC^b^** **µg/L**	**23.0** **11-43**	**27.0** **15-46**	**25.0** **14-42**	**14.9** **11-26**	**15.3** **10-22**	**20.2** **14-36**	**12.4** **5-21**	**15.3** **6-40**	**19.6** **4-42**

^a^ mean **±** 2 standard deviations (as reported by manufacturer); ^b^ median plus interquartile range (as reported by manufacturer); ^c^ pre-menopausal female; ^d^ post-menopausal female

## Conclusions

These results demonstrate the large between-person variability in serum bone markers and the differences between defined ‘healthy control’ ranges, this can be critical when assessing patients with bone disease. We suggest that it is therefore important for each laboratory to investigate the transferability of the quoted reference range to its own patient population based on equivalent standardised collection conditions, and where necessary determine its own ranges. We recognize that it is often difficult to recruit sufficient healthy volunteers but the widespread availability of automated bone marker assays now means that harmonisation of methods and specific reference ranges may be possible using well-characterised populations in larger cohorts.

